# Efficient Hydrolysis of Chitin in a Deep Eutectic Solvent Synergism for Production of Chitin Nanocrystals

**DOI:** 10.3390/nano10050869

**Published:** 2020-04-30

**Authors:** Shu Hong, Yang Yuan, Kaitao Zhang, Hailan Lian, Henrikki Liimatainen

**Affiliations:** 1 College of Materials Science and Engineering, Nanjing Forestry University, Nanjing 210037, China; hongshu.320@163.com; 2 Fibre and Particle Engineering Research Unit, University of Oulu, P.O. Box 4300, 90014 Oulu, Finland; yuan1118927@163.com (Y.Y.); Kaitao.Zhang@oulu.fi (K.Z.)

**Keywords:** chitin, nanocrystals, deep eutectic solvent, emulsion

## Abstract

A deep eutectic solvent (DES) derived from ferric chloride hexahydrate and betaine chloride (molar ratio of 1:1) was used as hydrolytic media for production of chitin nanocrystals (ChNCs) with a high yield (up to 88.5%). The synergistic effect of Lewis acid and released Brønsted acid from betaine hydrochloride enabled the efficient hydrolysis of chitin for production of ChNCs coupled with ultrasonication with low energy consumption. The obtained ChNCs were with an average diameter of 10 nm and length of 268 nm, and a crystallinity of 89.2% with optimal synthesis conditions (at 100 °C for 1 h with chitin-to-DES mass ratio of 1:20). The ChNCs were further investigated as efficient emulsion stabilizers, and they resulted in stable o/w emulsions even at a high oil content of 50% with a low ChNC dosage of 1 mg/g. Therefore, a potential approach based on a DES on the production of chitin-based nanoparticles as emulsifiers is introduced.

## 1. Introduction

Chitin, which consists of poly-β-(1-4)n-acetyl-D-glucosamine, is the second most abundant biopolymer in nature after cellulose. It can be obtained from renewable marine sources (i.e., sponges) [1] as well as different feedstock, such as crustacean shells [2,3,4,5], spiders [6,7,8] and insect cuticles [6], and some cell walls of fungi and diatoms [9], in which it plays an important role in the skeleton of these biological structures. Chitin has many appealing characteristics, such as nontoxicity, sustainability, biocompatibility, and biodegradability, and it has attracted notable attention during recent years. It is already applied in multiple sectors, such as food [10], medical [11], cosmetics [12],agriculture [13], drug release [14], tissue engineering [15,16,17], environment [18,19] and extreme biomimetics [20,21], as commercial products.

Numerous studies have been conducted to extract or optimize the extraction conditions of chitin from a variety of sources [3,4,5,6,22,23], but only few solvents can be used for dissolving or dispersing chitin for further processing [24]. However, when the size of chitin is reduced to nanoscale, it readily disperses in water under acidic or even neutral conditions [25]. Chitin nanocrystals (ChNCs) have a rod-like micromorphology, large surface area, low density, and outstanding mechanical toughness. Because of these advantageous features, ChNCs have been used as a reinforcement of polymer composites [26], scaffolds [27], emulsifiers [28], antimicrobial agents [29], and so on. Generally, ChNCs are prepared through acid hydrolysis (HCl), surface partial deacetylation or 2,2,6,6-tetramethylpiperidine-1-oxyl (TEMPO)-mediated oxidation, followed by mechanical disintegration [26,30,31].

Deep eutectic solvents (DESs) are self-associated liquid mixtures that typically consist of hydrogen bond donor (HBD) and hydrogen bond acceptor (HBA) pairs that form a system with a significantly lower melting point than each of the individual components. DESs are often considered ionic liquid (IL) analogues, exhibiting similar characteristics, such as low vapor pressure and good solvent capability [32]. However, DESs are commonly inexpensive, commercially available, and easier to prepare [25,33] compared with ILs. Because DESs can efficiently interact with the hydrogen bond systems of carbohydrates and biomasses, they have been studied as sustainable and customizable chemicals for the green processing of polysaccharides [4,34]. For example, DES composed of choline chloride and oxalic acid dihydrate was found to be efficient in hydrolyzing amorphous regions of cellulose for the production of cellulose nanocrystals (CNCs) [35]. The addition of ferric chloride further enhanced the liberation of CNCs without any mechanical treatment [36]. Moreover, acidic DESs have been reported to act as effective medium for the fabrication of ChNCs, and DESs of organic acid and choline chloride was used for the synthesis of ChNCs [37]. In addition, choline chloride and zinc chloride DES were used as reaction media and catalysts for the production of esterified ChNCs in a one-step reaction [25]. The obtained ChNC suspensions were stable over a wide pH range. As a kind of designable solvent, and based on these studies, it is highly possible to find out a new kind of DES as efficient hydrolytic medium of chitin for production of ChNCs. 

Previously, ChNCs displayed capability of forming stable emulsions, even at an extremely low concentration without any modification [28,38,39,40]. Natural and nontoxic emulsifiers are highly desired as many synthetic surfactants need to be replaced because of more stringent legislations, e.g., in European countries [41]. Bio-based particles are attractive in replacing synthetic surfactants, which possess potential toxic and harmful environmental effects [41,42]. In the present work, we demonstrated a new DES composed of ferric chloride hexahydrate and betaine hydrochloride (FeBCl) as a pretreatment of alpha chitin for the nanofibrillation of non-derivatized ChNCs with ultrasonication. The ChNCs were analyzed with Fourier transform infrared spectroscopy (FT-IR), transmission electron microscope (TEM), X-ray powder diffractometry (XRD), and thermogravimetric analysis (TGA). Moreover, the performance of ChNCs as efficient emulsion stabilizers in an aqueous system of soybean oil was addressed.

## 2. Materials and Methods 

### 2.1. Materials

Alpha (α)-chitin from shrimp shells (particle grade), soybean oil (from soybean), and ferric chloride hexahydrate (regent grade, purity >98%) were purchased from Sigma-Aldrich (Steinheim, Germany). Absolute ethanol and 0.1 M HCl were obtained from VWR (Helsinki, Finland) and betaine hydrochloride (purity >98%) from TCI (Eschborn, Germany). All chemicals were used without any further purification. Deionized water (DI water, whose conductivity is around 2.0 μS/cm) was used throughout the experiments.

### 2.2. Pretreatment of Chitin with DES

DES was first prepared by mixing betaine hydrochloride and ferric chloride hexahydrate with a molar ratio of 1:1 in a beaker. Then, the mixture was transferred to an oil bath (80 °C) and was continuously mixed with a magnetic stirrer until it formed a homogeneous solution. Next, the desired amount of chitin was added in DES with a mass ratio of 1:20 at desired temperatures (70, 80, 90, and 100 °C) and time (1, 2, 3, and 4 h) (Table 1). After the DES treatment, the mixture was let stand at room temperature for 5 min and ethanol was added, after which the suspension was centrifuged at 8000 rpm for 5 min (Beckman Coulter, Avanti J-26 XPI, Fullerton, CA, USA) using a JA-25.50 rotor. Chitin sediment was resuspended in 0.1 M HCl and centrifuged again until turbid suspension was obtained. The suspension was then collected and transferred in a dialysis tubing cellulose membrane (typical molecular weight cut-off = 14 kDa; Sigma-Aldrich, Steinheim, Germany) until it became neutral.

### 2.3. Liberation of ChNCs

The diluted aqueous chitin suspension (1.0 wt %) was treated in an ultrasonic processor (UP400S, Hielscher, Teltow, Germany; power max = 400 W) equipped with a titanium sonotrode with output power of 320 W for 5 min. The treatment was performed using a pulse of 0.5/0.5 s on/off and amplitude of 80% at a 24 kHz frequency.

### 2.4. Characterization of ChNCs

FT-IR spectra was recorded on a Bruker Vertex 80v spectrometer (Ettlingen, Germany) from freeze-dried ChNC samples. The spectra were collected at ambient conditions from an accumulation of 40 scans at a 2 cm^−1^ resolution over the regions of 600–4000 cm^−1^.

The crystalline structure of the chitin samples was characterized using a powder XRD (Bruker D8 ADVANCE instrument, Karlsruhe, Germany) with CuKα radiation (λ = 1.5406) (40 kV, 30 mA). The scan range was from a 2θ angle of 5 to 55 °C with a scanning speed of 4 °C per minute. The crystallinity index (CrI; %) was calculated in accordance with Equation (1) [43].

(1) $$CrI_{110}=\left[\frac{I_{110}-I_{am}}{I_{110}}\right]*100\%,$$

where *I*_110_ is the maximum intensity at 2*θ* ≅ 19.2 °C and *I_am_* is the intensity of an amorphous diffraction at 2*θ* ≅ 16 °C.

The micromorphological features of ChNCs were analyzed with a Tecnai G2 Spirit transmission electron microscope (TEM; FEI Europe, Eindhoven, Netherlands). A droplet of 0.1 mg/mL ChNC suspension was dosed on a carbon-coated copper grid and was left to dry for 1 min. A drop of stain (7 µL of uranyl acetate, 2% w/v) was added to the grid and let stand for 1 min, after which the TEM grid was dipped in a beaker containing DI water. The grid was then allowed to fully dry in air and in the sample case at least 12 h prior to imaging with the TEM.

TGA and derivative thermogravimetric analysis (DTG) were carried out using a Mettler Toledo TGA 2 SF/1100 apparatus (Mettler Toledo, New York, NY, USA). Approximately 3 mg samples were dosed in an aluminum pan and analyzed with a heating rate of 10 °C min^−1^ from 30 to 600 °C under nitrogen atmosphere with a gas flow rate of 30 mL/min.

### 2.5. ChNC-Stabilized Pickering Emulsions

Soybean oil emulsions possessing an o/w ratio from 20/80 to 50/50 and a ChNC concentration of 0.05–0.5 wt % were formed using ultrasonication with a titanium sonotrode for 2 min with a pulse sequence of 0.5/0.5 s on/off and amplitude of 80% operating at 24 kHz frequency and 320 W power processor (UP400S, Hielscher, Teltow, Germany). The oil droplet size was measured using a laser diffraction particle size analyzer (LS 13 320; Beckman Coulter, Indianapolis, IN, USA). The morphology of the oil droplet was observed and imaged using a Leica MZ LIII stereomicroscope (Leica Microsystems Ltd., Heerbrugg, Switzerland), with a 10× objective lens from a drop of emulsion dipped onto a microscope slide.

## 3. Results and Discussion

### 3.1. Effect of DES Treatment Conditions on the Appearance and Yield of ChNCs

Betaine hydrochloride is an acidic form of betaine and can be used as a supplemental source of hydrochloric acid during the hydrolysis reaction of chitin. It is natural and biodegradable and can be found in sugar beets and other plants. On the contrary, ferric chloride was found to be an efficient catalyst for assisting the hydrolysis reaction of cellulose to fabricate CNCs even without any further mechanical treatment [36,44]. In this study, chitin was treated with a DES system of betaine hydrochloride and ferric chloride hexahydrate. After the solvent treatment, a highly hydrophilic chitin slurry, which was difficult to wash using a filtration method because of the blocking of the filter paper, was formed. This phenomenon indicated an increase in the surface area of chitin and disintegration of chitin to its smaller constituents as previously found with HCl hydrolysis and partially deacetylated chitin [31,45]. The sonication further promoted the disintegration of chitin and resulted in ChNC suspensions.

**Figure 1:**
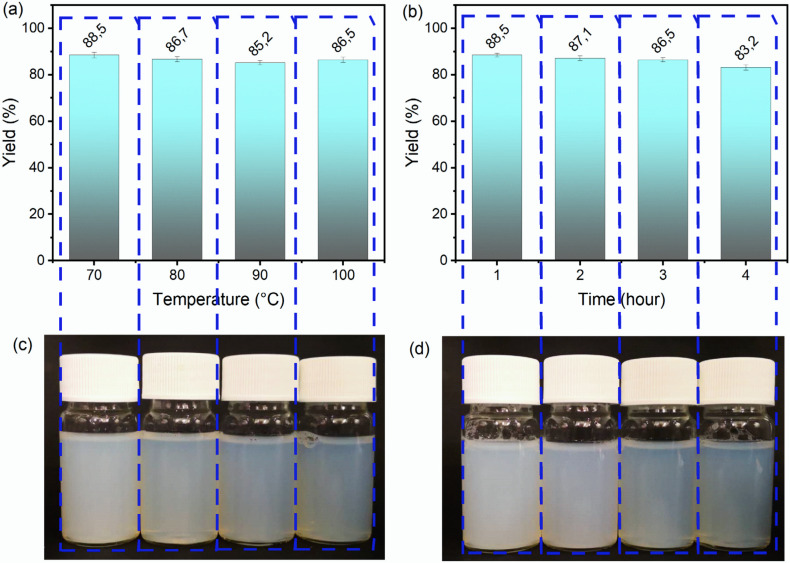
Mass yield of ChNCs after DES treatment (and ultrasonication) at different temperatures (3-h treatment) (**a**) and for different reaction time (at 100 °C) (**b**) and the appearance of the corresponding suspensions (**c**,**d**).

First, the influence of the reaction temperature of DES treatment on the mass yield and appearance of ChNC suspensions was examined. Temperature from 70 to 100 °C for a fixed reaction time of 3 h was used, as shown in Figure 1a. The appearance of corresponding ChNC suspensions (0.4 wt %) is shown in Figure 1c. The yield of ChNCs slightly decreased as a function of temperature, presumably due to the over-hydrolysis of the chitin molecule chain under an elevated temperature. However, the yields were high, being >85 wt % with all treatment times. The visual appearance of ChNC suspensions gradually turned from milky to more transparent with the increased reaction temperature, ascribing to a more efficient liberation of individual ChNCs [46]. To ensure an efficient hydrolysis of chitin, the reaction temperature was fixed at 100 °C for further experiments, and the role of reaction time on the ChNC production was studied (Figure 1b,d). The yield of ChNCs slightly decreased from 88.5 to 83.2 wt % as expected with the prolonged reaction time (from 1 to 4 h). Moreover, the ChNC suspensions turned from milky white to transparent with a blue color with an increase in the temperature.

The highest yield of 88.5 wt % was obtained at 70 °C with a treatment time of 1 h using the FeBCl DES and ultrasonication for 5 min. For the traditional HCl hydrolysis method, the yield has been reported to range from 40% to 86% [47,48,49]. The typical concentration of HCl has been 3 M, and the reaction temperature and reaction time have varied between 80 and 104 °C and 1.5–6 h, respectively [50]. The ultrasonication processing time has in turn typically ranged from 2.5 to 20 min [50]. Normally, the hydrolysis time and temperature depend on the chitin source [50]. For instance, an increased reaction was needed with shrimp shells (up to 6 h at 104 °C) [51,52]. Here, the maximum yield of ChNCs using the DES treatment was higher than that earlier reported with HCl hydrolysis, whereas the reaction conditions were similar or milder. Moreover, the DES system can likely be recycled after removing the washing liquid (ethanol) through evaporation. The residual ferric chloride removed from chitin with acid washing can potentially be transformed into high-value-added Fe(OH)_3_ by adding NaOH into the filtrate [36]. The only residues are nontoxic NaCl and betaine.

Recently, different strategies based on DESs were applied to treatment of chitin for fabrication chitin nanoparticles, including dissolving and regeneration [53], hydrolysis [37] and surface modification [25]. Choline chloride and thiourea were found to be able to dissolve chitin under heated condition [54], and this kind of DES was successfully used to dissolve and regenerate chitin for liberation of chitin nanofibers coupled with ultrasonication treatment for 40 min with the yield up to 86 wt % [53]. This might be due to the swelling of chitin in choline chloride and thiourea treatment; the regenerated chitin possesses a loosen molecular chain structure. The following ultrasonication treatment tends to peel off the nanofibrils from the bundles relatively easy. Choline chloride and organic acid based DESs were studied as hydrolysis medium of chitin for fabrication of ChNCs coupled with ultrasonication treatment and the yield is up from 87.5 wt % [37]. However, the mechanical treatment is more intense with output power of 1000 W and treatment time of 30 min. In current study, the output power is only 320 W, and the treatment time is 5 min, which showed a lower energy consumption during mechanical procedure. In another previously reported approach based on the DES of zinc chloride and choline chloride [25], the DES worked as the O-acetylation or esterification medium for the catalysis of chitin. Compared with zinc chloride, the toxicity of ferric chloride is lower than that of zinc chloride. Moreover, the processing conditions are much milder as the highest yield of ChNCs with zinc chloride and choline chloride was around 77 wt % with an ultrasonication treatment output power of 600 W and treatment time of 45 min. The higher energy demand indicates less efficient hydrolysis of chitin compared with that of FeBCl DES. Thus, FeBCl DES showed to be a promising and efficient new medium for the production of ChNCs with a high yield using less toxic chemicals.

### 3.2. Chemical Structure of ChNC

**Figure 2:**
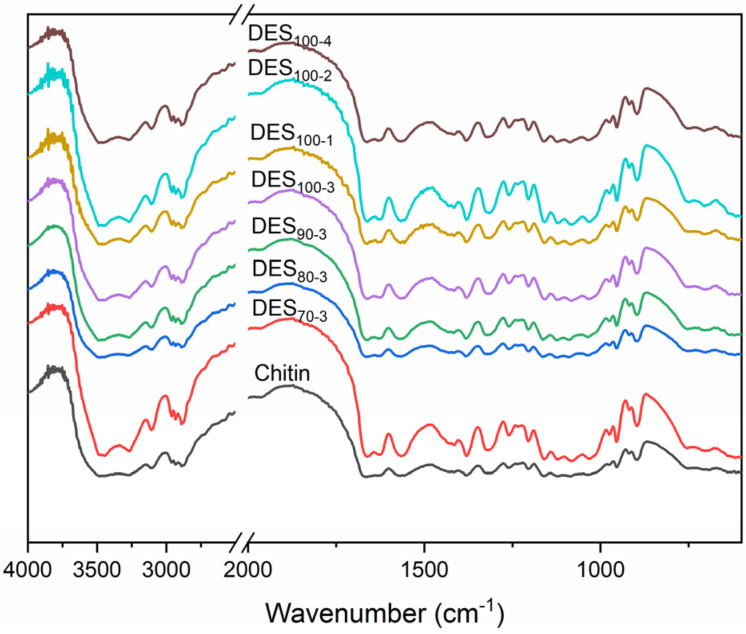
FT-IR spectra of pristine chitin and ChNCs prepared using DES under different reaction conditions.

The betaine hydrochloride molecule possesses a carboxyl group, which may react with the hydroxyl group in chitin and form an ester group. As a typical Lewis acid, ferric chloride is in turn able to act as a potential catalyst for the O-acylation reaction of chitin. FT-IR spectroscopy was used to analyze and confirm the chemical characteristics of chitin after the DES treatment (Figure 2). The broad bands at 3477 and 3264 cm^−1^ were ascribed to the O-H and N-H stretching, respectively, whereas the typical characteristic peaks of chitin were located at 1664, 1623, and 1565 cm^−1^ for amides I and II as reported also in previous studies [6,25,55]. Two sharp peaks of amide I confirmed that the structure of treated chitin samples was still α-chitin. The band around 1318 cm^−1^ was in turn assigned to the amide III band. Other bands in the spectra of pristine and DES-treated chitin samples were similar, demonstrating the unchanged structure of chitin. Due to the similar structure of the DES-treated chitin samples, only DES_70-3_, DES_100-1_, and DES_100-3_ were selected for further analysis to compare their physicochemical properties.

The acylation of carbohydrates seems to be an efficient approach to obtain well-dispersed nanoparticles from cellulose and chitin [46,56,57]. The grafting increases the charge density of carbohydrates, enhances the size reduction, and results in stable nanoparticle suspensions. Moreover, cationization reactions can promote the nanoparticle production from carbohydrates. For this purpose, betaine hydrochloride has been harnessed as a cationization chemical for lignocellulosic fibers [58]. However, the reaction needs specific conditions, e.g., the presence of a catalyst or high temperature. Therefore, the conditions used in the present work seem to not support the cationization of chitin by betaine hydrochloride. Presumably, the catalytic effect of ferric chloride is not as good as that in previously used DES systems based on triethylmethylammonium chloride and imidazole in the presence of *p*-toluenesulfonyl (tosyl) chloride [58]. Moreover, the highest temperature used here was only 100 °C, whereas the esterification reaction of betaine hydrochloric and hydroxyl group has previously reported to occur above 150 °C [59].

### 3.3. Morphology of ChNC

**Figure 3:**
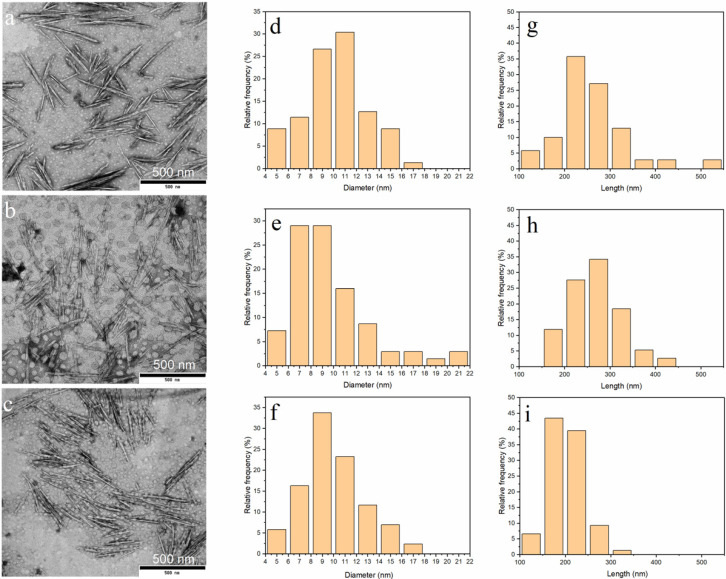
Transmission electron microscope (TEM) images and diameter and length distributions of ChNCs: (**a**,**d**,**g**) DES_70-3_, (**b**,**e**,**h**) DES_100-1_, and (**c**,**f**,**i**) DES_100-3_.

The TEM images of ChNCs stained with uranyl acetate are shown in Figure 3. The ChNCs exhibited a rod like morphology with a nanoscale diameter, being comparable with crystals obtained using hydrochloric acid as the hydrolytic medium [60]. In addition, some particles were observed to exist as larger bundles that consisted of oriented individual crystals. However, the appearance of DES_70-3_ and DES_100-1_ suspensions were milky like, and the micromorphology of these ChNCs was similar to that of DES_100-3_, which existed as a more transparent water suspension. However, obvious bundles of long fibers did not exist in the three samples, and chitin fibers (Figure S1) were disintegrated to nanoscale particles after the DES pretreatment with the assistance of ultrasonication. The average length and diameter of ChNCs were determined by analyzing at least 70 ChNCs of each sample from the TEM images (Table 2). The average diameter of all ChNCs was around 10 nm, being comparable to the ChNCs obtained with the TEMPO method and even smaller than those synthesized using hydrochloric acid hydrolysis in previous studies [30,50]. The average length of crystals varied from 268 to 201 nm depending on the reaction conditions and was shortest with DES_100-3_. The detailed distribution of the diameter and length of ChNCs is shown in Figure 3. The length distribution of ChNC DES_70-3_, DES_100-1_, and DES_100-3_ ranged from 120 to 530 nm, 160 to 440 nm, and 120 to 310 nm, respectively, indicating that the extended reaction time promoted the hydrolysis of chitin and led to a more even length distribution.

Previously, the DES of choline chloride and organic acids were used as an efficient hydrolytic solvent for chitin to result in ChNCs with a length of 257–670 nm and diameter of 42–49 nm [37]. The DES treatment with choline chloride and zinc chloride in turn produced O-acetylated ChNCs with a diameter and length ranging from 20 to 80 nm and 100 to 700 nm, respectively [25]. In the present study, ChNCs with smaller dimensions and higher aspect ratio were obtained. The high aspect ratio of ChNCs is beneficial when ChNCs are used, e.g., as reinforcements in composite materials.

### 3.4. Crystalline Structure and Thermal Properties of ChNC

**Figure 4:**
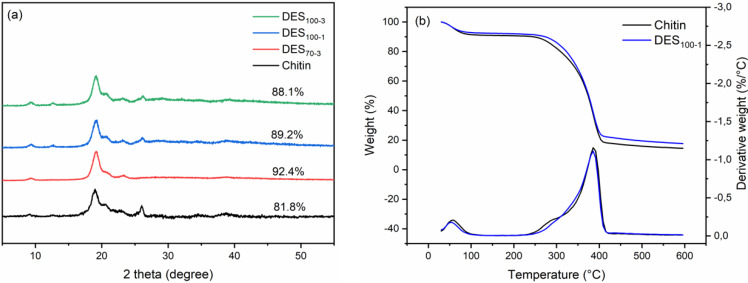
(**a**) XRD diffraction patterns of chitin and ChNCs prepared by DES and (**b**) TG and DTG curves of chitin and DES_100-1_ ChNCs.

The XRD patterns of the ChNCs and pristine chitin powder are presented in Figure 4a. The diffraction patterns show the characteristic peaks of chitin at 2θ of 9.2, 12.5, 19.2, 20.6, 23.2, and 26.3 °C attributed to the crystalline planes of (020), (021), (110), (120), (130), and (013), respectively [6,61]. These patterns correspond to the typical antiparallel crystalline structure of α-chitin, suggesting that the crystalline structure of the ChNCs was unaltered after the DES and ultrasonication treatments. These findings are also supported by the FT-IR results (Figure 2). Due to the unmodified structure of chitin, only the hydrolysis of the amorphous area occurred during the DES treatment. Therefore, the CrI values of the obtained ChNCs increased when compared with pristine chitin (Figure 4a). In a previous study, the obtained O-acetylation ChNCs showed a lower CrI value due to the introduced acetyl groups, which loosened the hydrogen-bonded crystalline structure of chitin [25]. As regards the high yield and energy consumption, among the conducted reaction conditions, DES_100-1_ was considered the optimal choice.

The thermal stability of nanoparticles is one of the key properties in its application. Therefore, the thermal stability of the DES_100-1_ and pristine chitin was studied using TGA and the DTG analysis, as shown in Figure 4b. The thermal stability of chitin is affected by its crystallinity, molecular weight, particle size, and source [3,4,6,62]. The start decomposition temperature (T_start_), onset decomposition temperature (T_onset_), and maximum weight-loss rate temperature (T_max_) were 198, 343, and 389 °C for pristine chitin and 210, 340, and 386 °C for DES_100-1_, respectively. The lower T_start_ value of pristine chitin was likely due to some impurities in the original chitin, e.g., protein residues. This was also suggested by the small peak in the DTG curve of pristine chitin at around 275 °C [63]. After the DES treatment, this peak disappeared, demonstrating the removal of impurity from chitin. In addition, the T_onset_ and T_max_ values of the samples were similar, indicating the unchanged thermal stability of ChNCs during the DES treatment. 

### 3.5. Pickering Emulsions Stabilized by ChNC

**Figure 5:**
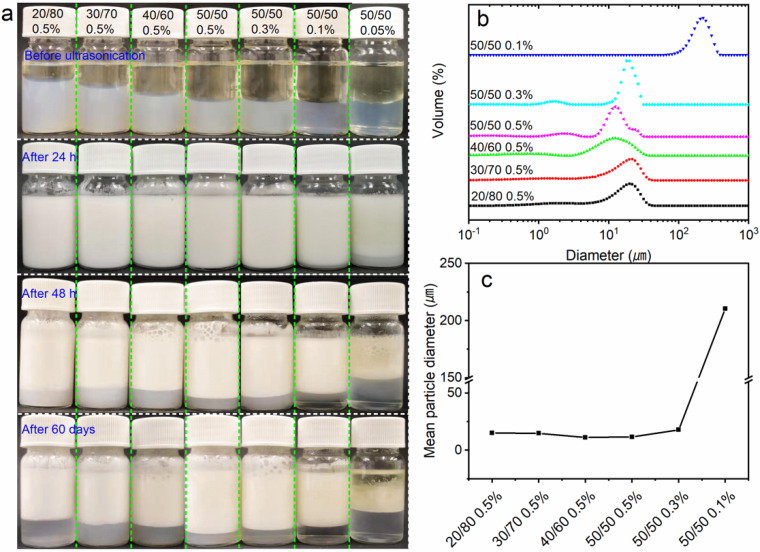
Appearance of soybean oil in water emulsions prepared by ChNCs (DES_100-1_) before and after sonication and after storage for 24 h, 48 h, and 60 days (**a**). Particle size distribution of emulsion droplets (**b**) and their mean diameter (**c**).

The general method used for preparing ChNCs was HCl hydrolysis coupled with mechanical treatment. When the pH of the ChNC suspension is around 3, the amino groups in chitin will all take positive integers, thus forming a stable suspension [38]. When the pH value turns to neutral or alkaline, the ChNC suspensions will immediately form sediment. Therefore, when using this type of ChNCs to prepare an emulsion, the pH of the emulsion is particularly important for stability and may limit its application. There are two main parameters affecting the emulsion process, i.e., pH and ionic strength, which is generally tuning by HCl or addition of NaCl [38]. Meanwhile, the obtained ChNCs suspensions in the current study were stable over a wide pH range even at neutral conditions. Here, ChNCs (DES_100-1_) were used to stabilize the emulsions derived from soybean oil and water using sonication, and the storage stability of emulsions with different o/w mass ratios and chitin concentrations was investigated at neutral condition without addition of NaCl. Figure 5a shows the emulsion before and after the ultrasonication treatment. All of the fresh emulsions formed smooth and creamy structures, except for the emulsion containing the lowest dosage of ChNC (0.05%, o/w 50/50). After a 24 h storage, emulsions having a ChNC concentration of 0.05% and 0.1% started to phase separate to form water (bottom) and creamy emulsion layers, whereas the other samples existed as uniform emulsions. After 48 h, all samples showed signs of phase separation, the effect being the strongest with the lowest ChNC dosages. This behavior was clearer after 60 days of storage.

The distributions of emulsion droplet diameter (Figure 5b) and mean droplet diameter (Figure 5c) were determined with a laser diffraction particle size analyzer. The average diameter was very similar regardless of the mass ratio of o/w. This result indicated that ChNCs efficiently adsorbed at the o/w interfaces and built up an oil droplet network stabilized by ChNC and surrounded by water as a continuous matrix [28]. The absorbed ChNCs were effective in preventing the aggregation of droplets even with a high oil mass fraction (up to 50%). By decreasing the ChNC concentration, the diameter of the droplet gradually increased. When the concentration was lower than 0.3%, the diameter significantly increased from several tens of micrometers to a few hundred micrometers (droplet morphologies are shown in Figure S2). This is ascribed to the low loading of ChNCs and reduced nanoparticle coverage on the o/w interface. Overall, stable emulsions with a moderate long-term stability were obtained even with low ChNC loading and when the droplets were of relatively larger in size. Thus, ChNCs can be considered to have potential, e.g., in food and cosmetic applications, because of their sustainable origin and nontoxicity.

## 4. Conclusions

ChNCs were successfully prepared through a DES formed from ferric chloride and betaine hydrochloride. DES hydrolysis with the assistance of a mild mechanical disintegration (ultrasonication) resulted in an efficient disintegration of chitin. The influence of different reaction conditions on the yield and characteristics of ChNC suspensions were analyzed. A high temperature and long reaction time resulted in the over-hydrolysis of chitin and led to a lower yield. Under the optimum reaction conditions (chitin-to-DES mass ratio of 1:20 at 100 °C for 1 h), the yield was up to 88.5%, which is higher than that previously reported with a hydrochloric acid treatment. The obtained ChNC suspensions exhibited an excellent stability after the mild ultrasonication treatment for 5 min. The chemical structure of ChNCs was unchanged during the DES treatment, and only the crystallinity of ChNCs increased due to the removal of the amorphous parts of chitin. The thermal stability of ChNCs obtained under optimal condition was very similar to that of pristine chitin, which is beneficial to its application, e.g., in thermoplastics.

The obtained ChNCs were successfully used as emulsifiers in an o/w system. The ChNCs can stabilize o/w emulsions containing 50% oil with a dose of 1 mg/g even when the droplets were relatively large in size. The increase in the concentration of ChNCs significantly reduced the size of emulsion droplets. Consequently, the ChNCs can potentially be used as green and nontoxic additives in food, cosmetic, and other functional products.

## Figures and Tables

**Table 1:** Conditions during the deep eutectic solvent (DES) treatment of chitin.

Samples	Mass Ratio	Temperature (°C)	Time (h)
DES_70-3_	1:20	70	3
DES_80-3_	1:20	80	3
DES_90-3_	1:20	90	3
DES_100-3_	1:20	100	3
DES_100-1_	1:20	100	1
DES_100-2_	1:20	100	2
DES_100-4_	1:20	100	4

**Table 2:** Dimensions of ChNCs prepared with DES under different reaction conditions.

Samples	Length (nm)	Diameter (nm)	Aspect Ratio
DES_70-3_	259 ± 75	10 ± 2.8	26
DES_100-1_	268 ± 57	10 ± 3.4	27
DES_100-3_	201 ± 39	10 ± 2.6	20

## Supplementary Materials

The following are available online at https://www.mdpi.com/2079-4991/10/5/869/s1. Figure S1: Morphology of pristine chitin; Figure S2: Droplet morphology of emulsions stabilized with different ChNCs concentrations (a) 0.5%, (b) 0.3%, and (c) 0.1%.
