# Supplementary material for: Efficient Hydrolysis of Chitin in a Deep Eutectic Solvent Synergism for Production of Chitin Nanocrystals
Source: Nanomaterials (Basel). 2020 Apr 30;10(5):869. doi: 10.3390/nano10050869 (PMC7279284; doi:10.3390/nano10050869)
Supplement: Supplementary file 1 [file nanomaterials-10-00869-s001.pdf]

Supplementary

Efficient Hydrolysis of Chitin in a Deep Eutectic Solvent Synergism for Production of Chitin Nanocrystals

Shu Hong [1,2], Yang Yuan [2], Kaitao Zhang [2], Hailan Lian [1,*] and Henrikki Liimatainen [2,*]

[1] College of Materials Science and Engineering, Nanjing Forestry University, Nanjing 210037, China; hongshu.320@163.com

[2] Fibre and Particle Engineering Research Unit, University of Oulu, P.O. Box 4300, 90014 Oulu, Finland; yuan1118927@163.com (Y.Y.); Kaitao.Zhang@oulu.fi (K.Z.)

* Correspondence: lianhailan@njfu.edu.cn (Ha.L.); Henrikki.Liimatainen@oulu.fi (He.L.); Tel.: +86-258-542-7531 (Ha.L.)

Received: 15 April 2020; Accepted: 27 April 2020; Published: date

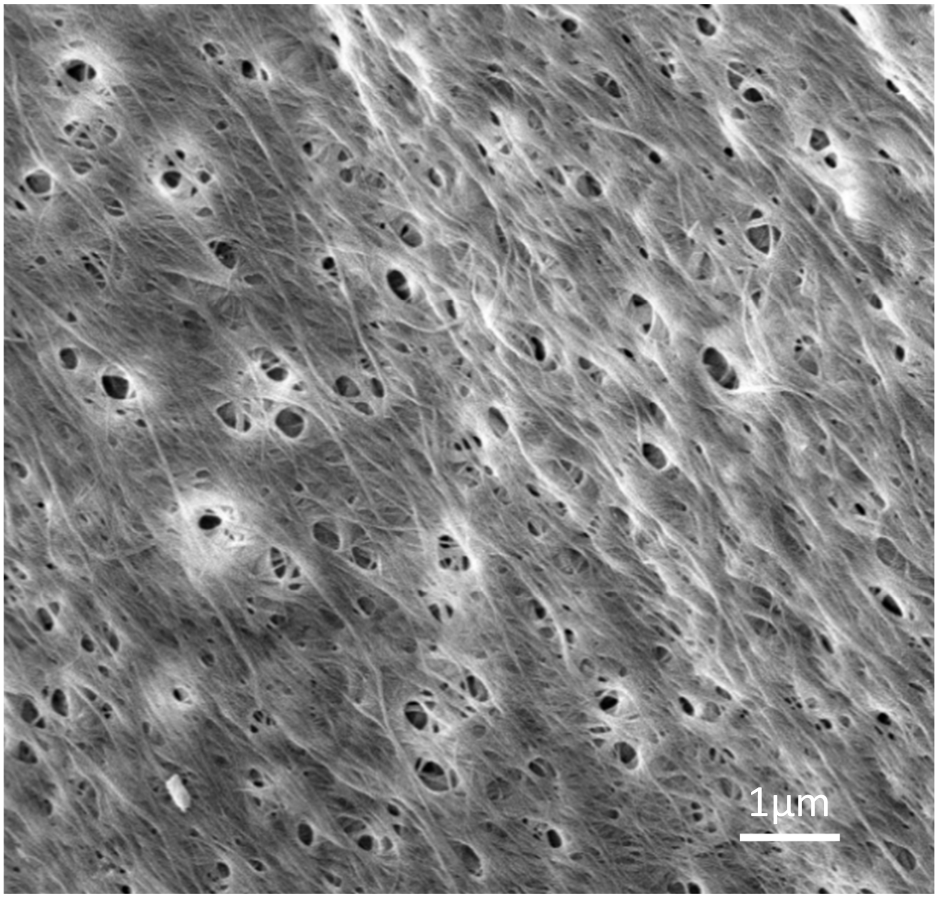

Figure S1. Morphology of pristine chitin.

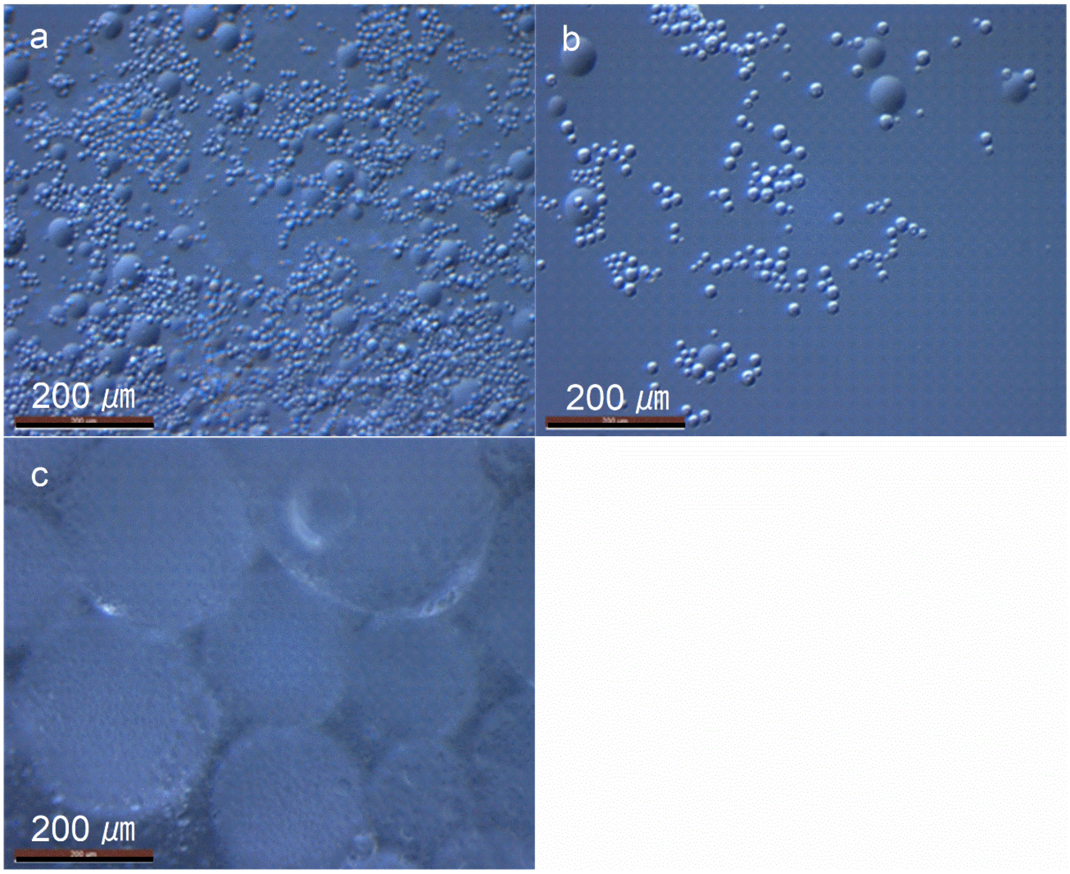

Figure S2. Droplet morphology of emulsions stabilized with different ChNCs concentrations (**a**) 0.5%, (**b**) 0.3%, and (**c**) 0.1%.
